# Diagnostic and therapeutic strategies for colorectal tumor with positive muscle‐retracting sign

**DOI:** 10.1002/deo2.278

**Published:** 2023-07-30

**Authors:** Hidenori Tanaka, Yudai Takehara, Shin Morimoto, Fumiaki Tanino, Noriko Yamamoto, Yuki Kamigaichi, Akiyoshi Tsuboi, Ken Yamashita, Takahiro Kotachi, Koji Arihiro, Shiro Oka

**Affiliations:** ^1^ Department of Gastroenterology Hiroshima University Hospital Hiroshima Japan; ^2^ Department of Anatomical Pathology Hiroshima University Hospital Hiroshima Japan

**Keywords:** colorectal endoscopic submucosal dissection, endoscopic ultrasonography, muscle‐retracting sign, pocket‐creation method, submucosal fibrosis

## Abstract

**Objectives:**

Endoscopic submucosal dissection (ESD) for colorectal tumors with positive muscle‐retracting (MR) sign often results in incomplete resection or discontinuation owing to the difficulty of submucosal dissection. The present study aimed to evaluate the usefulness of endoscopic ultrasonography (EUS) in diagnosing the MR sign and performing ESD using the pocket‐creation method (PCM).

**Methods:**

Thirty‐six cases of colorectal tumors with positive MR sign during ESD between January 2015 and December 2021 were retrospectively reviewed. Cases were divided into two groups: 1) the conventional method (CM) group, comprising 29 cases, and 2) the PCM group with seven cases, in which preoperative EUS and ESD using PCM were performed. Treatment outcomes were compared between the groups. The diagnostic yield of EUS for the MR sign was evaluated among large sessile tumors >20 mm in which preoperative EUS was performed.

**Results:**

Histologic diagnosis was adenoma or Tis carcinoma in 12 cases (33%), T1 carcinoma in 18 cases (50%), T2 carcinoma in four cases (11%), and unevaluable in two cases (6%). The sensitivity and specificity of the EUS‐MR sign for large sessile tumors were 87.5% and 83.3%, respectively. ESD was achieved in all cases in the PCM group, although it was discontinued in eight cases (28%) in the CM group. There were significant differences between the PCM and CM groups in en bloc resection (100% vs. 48%, *p* = 0.013) and R0 resection rates (71% vs. 31%, *p* = 0.049).

**Conclusion:**

The MR sign can be predicted by preoperative EUS, and ESD using PCM allows en bloc resection.

## INTRODUCTION

Colorectal endoscopic submucosal dissection (ESD) has been standardized by device development and establishment of technique.^1^, ^2^, ^3^, ^4^, ^5^, ^6^, ^7^, ^8^, ^9^, ^10^ However, submucosal fibrosis is a factor associated with the difficulties in performing an ESD.^11^, ^12^, ^13^, ^14^, ^15^ Large sessile tumors and laterally spreading tumors with large nodules sometimes exhibit severe submucosal fibrosis with the muscle layer pulled by the tumor, a condition termed muscle‐retracting (MR) sign.^16^ ESD for tumors with positive MR sign tends to result in incomplete resection or discontinuation because of the difficulty of submucosal dissection and perforation.^16^, ^17^ En bloc resection rate of ESD for tumors with positive MR sign was reported to be as low as 64%, and ESD had to be discontinued in the remaining 36% of cases.^16^ Therefore, ESD for this category of patients requires a high degree of endoscopic skill and techniques with ingenuity.

Endoscopic ultrasonography (EUS) is a useful modality that allows objective visualization of the colorectal wall; it is useful for the diagnosis of tumor invasion depth and dissectible submucosal space between the tumor‐invasive front and muscle layer.^18^, ^19^, ^20^, ^21^, ^22^, ^23^

The pocket‐creation method (PCM) is an ESD strategy that facilitates the maintenance of a thick submucosal layer by preventing leakage of the injected solution and providing good submucosal traction and scope operability.^24^, ^25^, ^26^, ^27^, ^28^

In this study, we aimed to evaluate the effectiveness of diagnostic strategies for MR sign of colorectal tumors via EUS; in addition, we aimed to perform ESD with PCM.

## METHODS

Of 1,330 cases of consecutive colorectal tumors with indications for ESD at Hiroshima University Hospital between January 2015 and December 2021, 36 cases of tumors with positive MR sign (2.7%) were retrospectively recruited for the study. The tumors were divided into two groups: the conventional method (CM) group, in which ESD using CM was performed between January 2015 and December 2019, and the PCM group, in which preoperative EUS and ESD using PCM were performed between January 2020 and December 2021. Preoperative EUS was performed by endoscopists with 20 or more cases of experience in performing EUS; ESD using PCM was performed by three expert endoscopists.

Treatment outcomes were compared between the groups. Scope operability was classified as poor, fair, or good, according to previous reports.^9^, ^13^ Postoperative bleeding was defined as any apparent bleeding, hematochezia, or >2 g/dL decrease in blood hemoglobin concentration compared with the preoperative level.^29^ R0 resection was pathologically identified as negative horizontal and vertical margins. Histologic diagnosis was categorized as adenoma, intramucosal (Tis) carcinoma, T1 carcinoma with submucosal invasion, and T2 carcinoma with invasion into the muscularis propria, according to the criteria of the Japanese Classification of Colorectal, Appendiceal, and Anal Carcinomas.^30^ Curative resection was defined as R0 resection of adenoma, Tis carcinoma, and T1 carcinoma with submucosal invasion depth <1,000μm satisfying all of the following characteristics: well/moderately differentiated or papillary carcinoma, no vascular invasion, and grade 1 budding.^31^

To evaluate the diagnostic yield of EUS for the MR sign, large sessile tumors >20 mm in diameter in which preoperative EUS was performed were recruited during the same period.

The study was conducted in accordance with the Declaration of Helsinki and the study protocol was approved by the Institutional Review Board of Hiroshima University (No. E2350).

### Indications for ESD

ESD was indicated in accordance with the current guidelines of the Japan Gastroenterological Endoscopy Society, which are as follows: lesions for which en bloc resection with snare endoscopic mucosal resection was difficult to apply, including laterally spreading tumor‐nongranular type, lesions showing a V_I_‐type pit pattern, carcinomas with shallow submucosal invasion, large depressed‐type tumors, and large protruded‐type lesions suspected to be carcinoma; mucosal tumors with submucosal fibrosis; sporadic tumors in conditions with chronic inflammation, such as ulcerative colitis; and local residual or recurrent early carcinomas after endoscopic resection.^32^, ^33^

### EUS procedure

EUS was performed in lesions in which submucosal invasion was suspected by endoscopic observation with white light imaging, magnified image‐enhanced imaging such as narrow‐band imaging, and chromoendoscopy. The procedure was also performed in large sessile tumors and laterally spreading tumors with large nodule lesions to predict the MR sign from January 2020.

**FIGURE 1:**
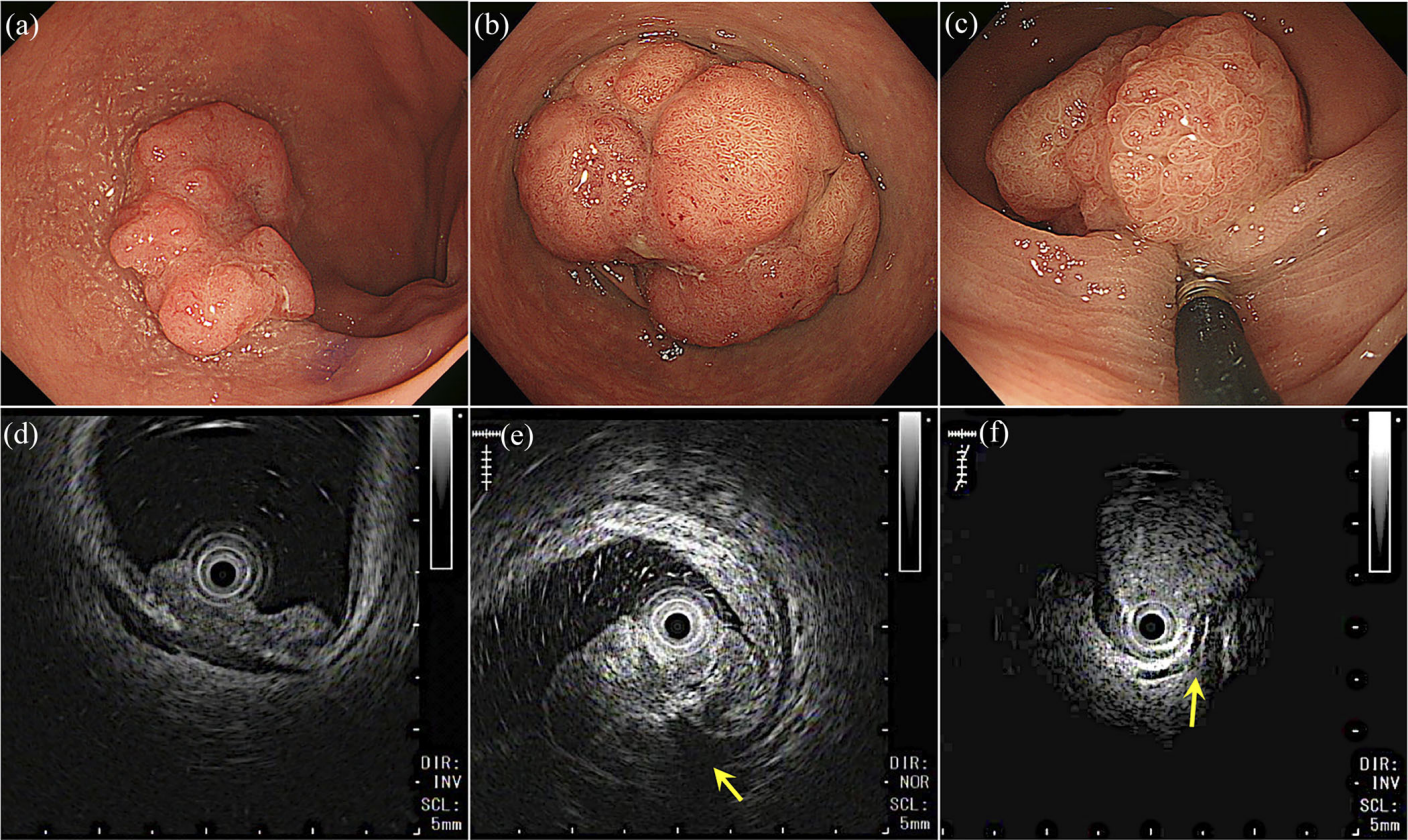
Images of the endoscopic ultrasonography muscle‐retracting (EUS‐MR) sign. Endoscopic images (a–c) and corresponding endoscopic ultrasonography images (d–f). Although the tumor is invading the low echoic layer corresponding to the muscle layer, the EUS‐MR sign is negative because the muscle layer is stretched (d). The EUS‐MR sign is positive when the low echoic layer corresponding to the muscle layer is pulled toward the mucosal lesion (yellow arrow e, f).

After the lumen was filled with saline, the lesion was scanned using a 20 MHz mini‐probe (UM‐DP20‐25R; Olympus). The colorectal wall was visualized as a five‐layered structure on EUS images. A lesion was diagnosed as a positive EUS‐MR sign when the fourth low echoic layer corresponding to the muscle layer was pulled toward the mucosal lesion (Figure 1). This was distinguished from a deep submucosal or more invasive carcinoma in which the tumor was invading the third high echoic or the fourth low echoic layers, but the fourth low echoic layer was stretched.

### ESD procedure

A colonoscope, either PCF‐Q260AZI (Olympus) or PCF‐H290TI (Olympus) was used. The ST hood (FUJIFILM, Tokyo, Japan) or its short type (FUJIFILM) was attached to the tip of the colonoscope. In addition, 0.4% sodium hyaluronate (Muco Up; Boston Scientific) diluted twice with 10% glycerin solution was used for submucosal injection.

**FIGURE 2:**
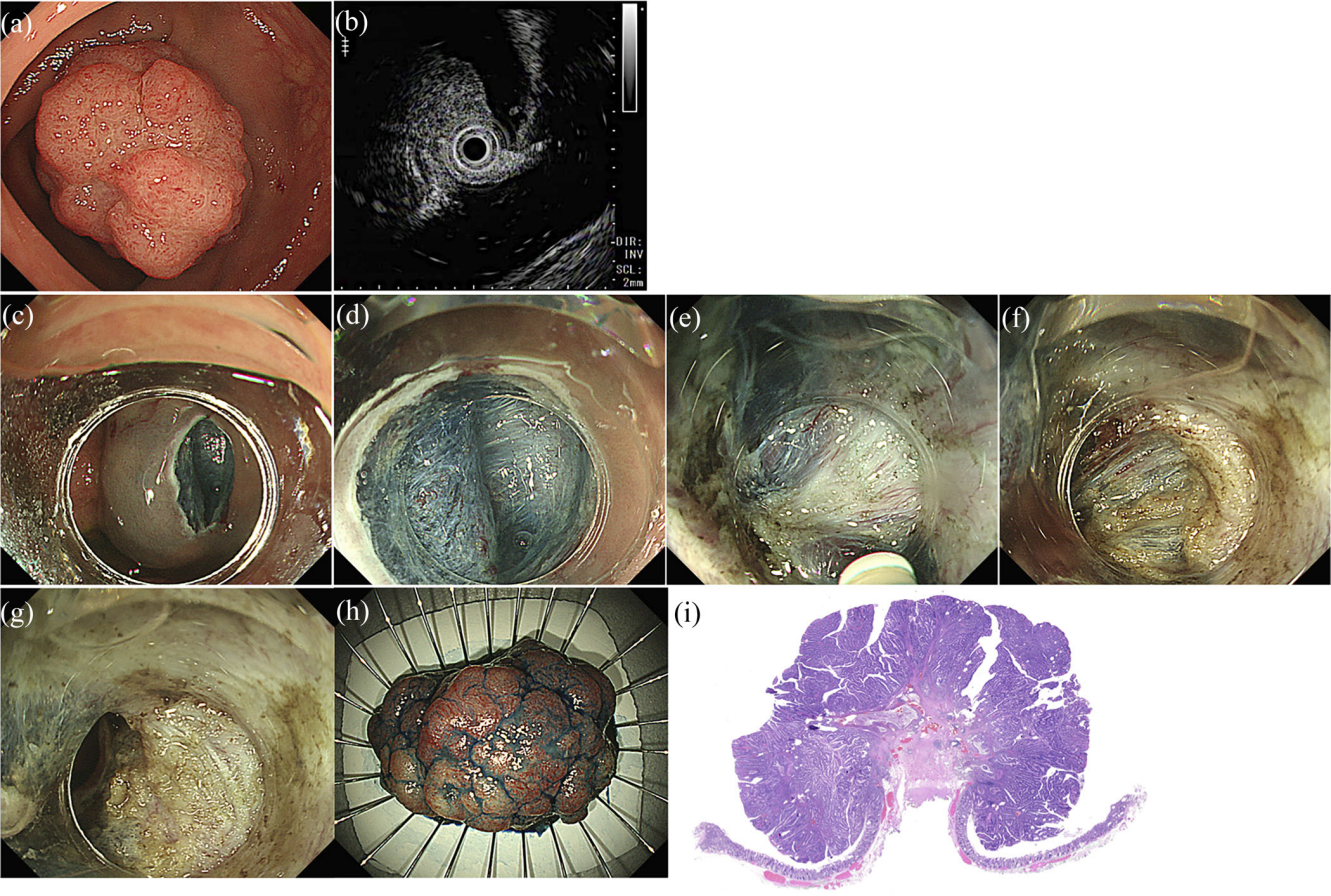
Large sessile tumor in the rectosigmoid colon (a). Endoscopic ultrasonography shows the muscle layer is pulled toward the mucosal lesion (b). En‐bloc resection is achieved without perforation by endoscopic submucosal dissection using the pocket‐creation method (c–g). The procedure time is 95 min. Histologic findings show submucosal invasive carcinoma (invasion depth 6000μm) with a negative vertical margin (h, i).

In the CM group, an initial mucosal incision of the anal side was made approximately one‐quarter or more of the circumference using DualKnife (Olympus) or DualKnife J (Olympus). After submucosal dissection was performed on both incised mucosal edges, additional mucosal incision and submucosal dissection were repeated in the same manner and finally reached the oral edge. ITknife nano (Olympus) and snare were used, as appropriate. In the PCM group, PCM was performed as described previously.^27^ The initial mucosal incision of the anal side was made approximately 20 mm in length and 10–15 mm away from the anal edge of the tumor. Submucosal dissection was performed by making a pocket via insertion of the tip of the scope into the submucosal layer. When severe fibrosis with the MR sign was recognized, dissection of the peripheral non‐fibrotic submucosa was performed in advance, the remaining exposed fibrosis was dissected by the tip of the needle, and the same procedure was repeated until the lesion and retracted muscle layer were separated. After submucosal dissection in the pocket was almost complete, an additional mucosal incision was made and dissection was performed to connect the pocket and peripheral mucosal incision (Figure 2).

### Statistical analysis

JMP version 16.0.0 (SAS Institute Inc.) was used for statistical analysis. Continuous variables were analyzed using the student's t‐test or Mann–Whitney U test and qualitative variables were analyzed using Pearson's chi‐squared test. *p*‐Values <0.05 were considered statistically significant.

## RESULTS

Clinicopathological features of colorectal tumors with positive MR sign included in the study are shown in Table 1. The mean tumor size was 43 mm in diameter, and tumor macroscopic type was 0‐Is in 18 cases (50%), 0‐Isp in 12 cases (33%), and 0‐IIa+Is in six cases (17%). Histologic diagnosis was adenoma or Tis carcinoma in 12 cases (33%), T1 carcinoma in 18 cases (50%), and T2 carcinoma in four cases (11%). Accurate histologic diagnosis was impossible owing to thermal denaturation and necrosis of the lesion in two cases in which ESD was discontinued and surgical resection was performed later.

**TABLE 1:** Clinicopathological features of colorectal tumors with a positive muscle‐retracting sign.

Variables	MR sign (+) *n* = 36
Age, years, mean ± SD	68 ± 14
Sex, male (%)	25 (69)
Tumor location (%)	
Right‐sided colon	14 (39)
Left‐sided colon	17 (47)
Rectum	5 (14)
Tumor size, mm, mean ± SD	43 ± 15
Macroscopic type (%)	
0‐Is	18 (50)
0‐Isp	12 (33)
0‐IIa+Is	6 (17)
Histology (%)	
Adenoma/Tis carcinoma	12 (33)
T1 carcinoma	18 (50)
T2 carcinoma	4 (11)
Unevaluable	2 (6)

Abbreviations: MR, muscle‐retracting; SD, standard deviation.

Clinicopathological features and ESD outcomes are presented in Table 2. There were no cases in which initial surgery was performed due to a positive EUS‐MR sign. ESD was achieved in all cases in the PCM group, whereas it was discontinued in eight (28%) cases in the CM group. Snare was used for rescuing and finishing the procedure in 10 (34%) cases in the CM group. There were significant differences in en bloc resection between the PCM and CM groups (100% vs. 48%, *p* = 0.013), R0 resection (71 % vs. 31%, *p* = 0.049), and curative resection rates (71% vs. 24%, *p* = 0.017). Two cases of non‐R0 resection in the PCM group were due to a positive vertical margin of T1 carcinoma.

**TABLE 2:** Clinicopathological features and outcomes.

Variables	CM *n* = 29	PCM *n* = 7	*p*‐value
Tumor location (%)			0.28
Right‐sided colon	12 (41)	2 (29)	
Left‐sided colon	12 (41)	5 (71)	
Rectum	5 (17)	0 (0)	
Tumor size, mm, mean ± SD	43 ± 17	41 ± 9	0.79
Growth type (%)			0.049
0‐Is	17 (59)	1 (14)	
0‐Isp	7 (24)	5 (71)	
0‐IIa+Is	5 (17)	1 (14)	
Histology (%)			0.44
Adenoma/Tis carcinoma	9 (31)	3 (43)	
T1a carcinoma	14 (48)	4 (57)	
T1b carcinoma	4 (14)	0 (0)	
Unknown	2 (7)	0 (0)	
Procedure time, min, mean ± SD	175 ± 114	152 ± 75	0.84
Scope operability (%)			0.34
Good	4 (14)	1 (14)	
Fair	7 (24)	0 (0)	
Poor	18 (62)	6 (86)	
Use of snaring (%)	10 (34)	0 (0)	0.07
ESD achievement (%)	21 (72)	7 (100)	0.12
En bloc resection (%)	14 (48)	7 (100)	0.013
R0 resection (%)	9 (31)	5 (71)	0.049
Curative resection (%)	7 (24)	5 (71)	0.017
Adverse event (%)			
Intraoperative perforation	9 (31)	1 (14)	0.37
Delayed perforation	1 (3)	0 (0)	0.62
Postoperative bleeding	0 (0)	0 (0)	‐

Abbreviations: CM, conventional method; ESD, endoscopic submucosal dissection; PCM, pocket‐creation method; SD, standard deviation;

**FIGURE 3:**
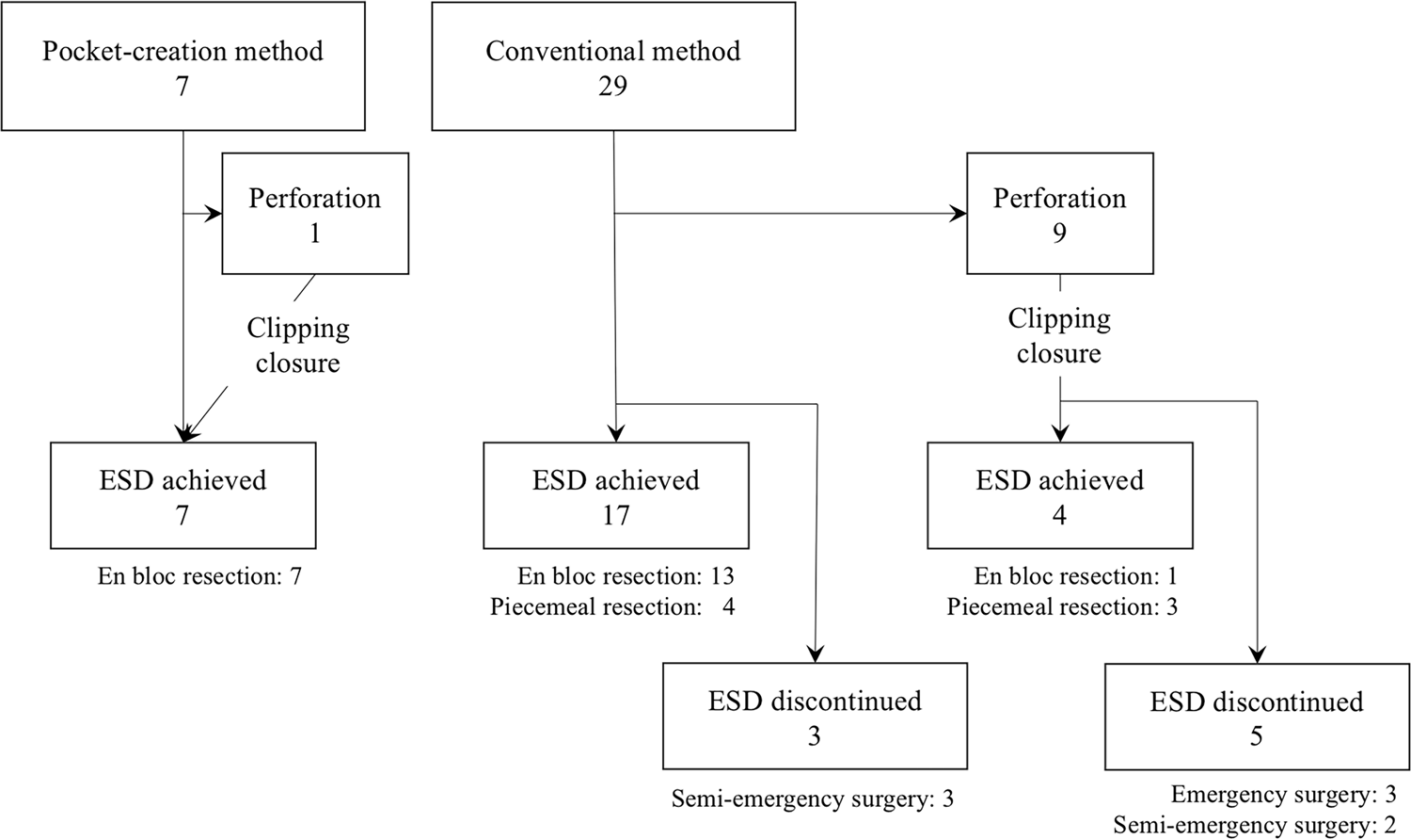
Clinical course during and after endoscopic submucosal dissection.

Clinical course during and after ESD is shown in Figure 3. In the PCM group, intraoperative perforation occurred in one case, and the perforation was closed by clipping, and en bloc resection was achieved in all cases. In the CM group, intraoperative perforation occurred in nine cases, and the perforations were closed by clipping. However, ESD was discontinued, and surgery was required owing to the difficulty in continuing submucosal dissection in five cases. In four cases in which ESD was achieved, en bloc resection was achieved in only one. Of the 20 cases without perforations in the CM group, ESD was discontinued in three cases owing to difficulty in continuing submucosal dissection.

The sensitivity and specificity of the EUS‐MR sign for sessile tumors >20 mm in diameter were 87.5% and 83.3%, respectively (Table 3).

**TABLE 3:** Diagnostic yield of endoscopic ultrasonography for muscle‐retracting sign.

	MR sign during ESD	
EUS‐MR sign	Positive	Negative
Positive	7	2
Negative	1	10

Abbreviations: ESD, endoscopic submucosal dissection; EUS, endoscopic ultrasonography; MR, muscle‐retracting.

## DISCUSSION

The present study showed that preoperative strategies EUS and PCM, respectively, facilitated prediction and en bloc resection of colorectal tumors with positive MR sign.

ESD for lesions with positive MR sign often results in failure because the submucosal flap cannot be made, integrated fibrosis and muscle layer are faced, and an appropriate submucosal dissection line is not visualized and set. Toyonaga et al. reported that en bloc resection was achieved in 18 of 28 cases (64%) with positive MR sign by ESD, and ESD was discontinued and switched to surgical resection in 10 cases,^16^ indicating that the ESD outcomes for lesions with positive MR sign were poor. The cause of the MR sign is considered to be a desmoplastic reaction to cancer invasion and fibrosis caused by the mechanical force generated between the submucosa and muscular layer.^16^ Most lesions with positive MR sign are invasive carcinomas, as a previous report showed that 22 of 28 cases (79%) were T1 carcinomas^16^; this present study showed similar findings, with 22 of 36 cases (61%) being T1 or T2 carcinomas. However, there were some adenomas and Tis carcinomas with positive MR sign. Therefore, it is better to achieve a minimally invasive endoscopic resection and avoid excessive surgery, if possible, although additional surgery may be required as a result of non‐curative resection.

In PCM, the muscle layer is stretched by the scope in the narrow pocket, and the thickness of the submucosal layer by the injected solution is maintained, allowing easy visualization and setting of an appropriate dissection line. In addition, good traction is obtained by the narrow pocket, as well as the tumor weight, allowing effective submucosal dissection. These advantages are especially useful for lesions with severe fibrosis and, therefore, for lesions with positive MR signs. The distance from the tumor edge to the initial mucosal incision should be longer than usual, approximately 10–20 mm, to successfully create a pocket. In the severe fibrotic area, it is important to perform shallow dissection tracing of the surface without deeply pushing the needle to avoid perforation. Yamashina et al. reported good outcomes, as R0 resection was achieved in five of six (83%) large sessile tumors with positive MR sign by ESD using PCM.^34^ In the present study, although the outcomes of ESD by CM were poor, with 31% perforation, 48% en bloc resection, 24% piecemeal resection, and 28% discontinuation rates, ESD performed using PCM had good outcomes, with 100% en bloc resection and 71% R0 resection rates. However, it should be noted that six of seven cases had poor scope operability even with PCM, and dissecting a severe fibrotic area requires high endoscopic skills even with the use of PCM; therefore, it should be performed by endoscopists with sufficient experience.

Although the MR sign is observed during submucosal dissection and is difficult to diagnose preoperatively, protruded‐type tumors show higher MR sign‐positive rates compared to flat‐type tumors.^16^, ^17^ Nevertheless, it is present in at most 41% of sessile tumors^16^ and 21% of protruded‐type tumors.^17^ We demonstrated that the sensitivity and specificity of the EUS‐MR sign for sessile tumors >20 mm in diameter were 87.5% and 83.3%, respectively. EUS diagnosis of tumor invasion depth is sometimes challenging in protruded tumors and tumor position on the folds or flexures^35^, ^36^ because the probe must be applied horizontally to the mucosa. However, the EUS‐MR sign can be observed easily by placing the probe on the protruding part of the tumor; this way, it is not affected by the attenuation of echoes. There were a few false positive and false negative cases. Capturing the inclined muscle layer and the inability to capture the appropriate part of the true MR sign might be the cause of the false positives and false negatives, respectively.

Based on the results of this study, we recommend that PCM should be used for ESD for large sessile tumors and laterally spreading tumors with large nodules on suspicion of the MR sign. In addition, we suggest that preoperative EUS should be performed to predict MR sign and select an appropriate endoscopist with a high level of skill and sufficient experience.

This study had some limitations. First, this was a retrospective study conducted at a single center. Second, the number of cases was relatively small. Third, there was a possibility of a selection bias in that EUS was not performed in all cases. The decision to perform EUS depended on endoscopists. Fourth, ESD using PCM was performed by expert endoscopists; therefore, its acceptability among less experienced endoscopists is uncertain. The learning curve of ESD using PCM is also unknown. Verification through a prospective study is, therefore, required. Nevertheless, we believe that EUS helps in the preoperative diagnosis of the MR sign, and there is no disadvantage in performing PCM for lesions with positive EUS‐MR findings, even if the MR sign was not observed during ESD.

In conclusion, the MR sign can be predicted by preoperative EUS for large sessile tumors and laterally spreading tumors with large nodules, and en bloc resection can be achieved by ESD using PCM.

## CONFLICT OF INTEREST STATEMENT

None.
